# Ion Heating in Advanced Dielectric Barrier Discharge Ion Sources for Ambient Mass Spectrometry

**DOI:** 10.1021/jasms.3c00087

**Published:** 2023-05-26

**Authors:** Marcos Bouza, Ezaz Ahmed, Priscilla Rocío-Bautista, Sebastian Brandt, Joachim Franzke, Antonio Molina-Díaz, Juan F. García-Reyes, William A. Donald

**Affiliations:** † Analytical Chemistry Research Group, Department of Physical and Analytical Chemistry, University of Jaén, Campus Las Lagunillas, 23071 Jaén, Spain; ‡ School of Chemistry, University of New South Wales, Sydney, New South Wales 2052, Australia; § ISAS—Leibniz Institut für Analytische Wissenschaften, Bunsen-Kirchhoff-Str. 11, 44139 Dortmund, Germany

## Abstract

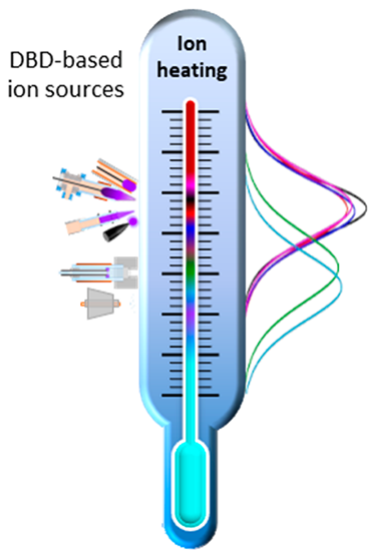

Dielectric barrier discharges (DBD) are highly versatile plasma sources for forming ions at atmospheric pressure and near ambient temperatures for the rapid, direct, and sensitive analysis of molecules by mass spectrometry (MS). Ambient ion sources should ideally form intact ions, as in-source fragmentation can limit sensitivity, increase spectral complexity, and hinder interpretation. Here, we report the measurement of ion internal energy distributions for the four primary classes of DBD-based ion sources, specifically DBD ionization (DBDI), low-temperature plasma (LTP), flexible microtube plasma (FμTP), and active capillary plasma ionization (ACaPI), in addition to atmospheric pressure chemical ionization (APCI) using para-substituted benzylammonium thermometer ions. Surprisingly, the average extent of energy deposited by the use of ACaPI (90.6 kJ mol^–1^) was ∼40 kJ mol^–1^ lower than the other ion sources (DBDI, LTP, FμTP, and APCI; 130.2 to 134.1 kJ mol^–1^) in their conventional configurations, and slightly higher than electrospray ionization (80.8 kJ mol^–1^). The internal energy distributions did not depend strongly on the sample introduction conditions (i.e., the use of different solvents and sample vaporization temperatures) or the DBD plasma conditions (i.e., maximum applied voltage). By positioning the DBDI, LTP, and FμTP plasma jets on axis with the capillary entrance to the mass spectrometer, the extent of internal energy deposition could be reduced by up to 20 kJ mol^–1^, although at the expense of sensitivity. Overall, the use of an active capillary-based DBD can result in substantially less fragmentation of ions with labile bonds than alternate DBD sources and APCI with comparably high sensitivity.

## Introduction

Ambient ionization mass spectrometry is beneficial for the simple, rapid, and direct analysis of small molecules from many different types of samples.^1,2^ Many applications have emerged for ambient ionization across multiple disciplines, including medical diagnostics and pharmaceuticals, forensics, and environmental science.^3^ Within the past two decades, over 70 different types of ambient ionization methods have been developed involving the use of plasmas,^4^ sprays,^5^ or lasers.^6^ A well-known category of plasma-based ionization methods involves the use of dielectric barrier discharges (DBDs).^7^ Such ion sources are highly tolerant of samples containing complex, heterogeneous mixtures and can be used to detect intact polar and nonpolar compounds with high sensitivity.

**Figure 1:**
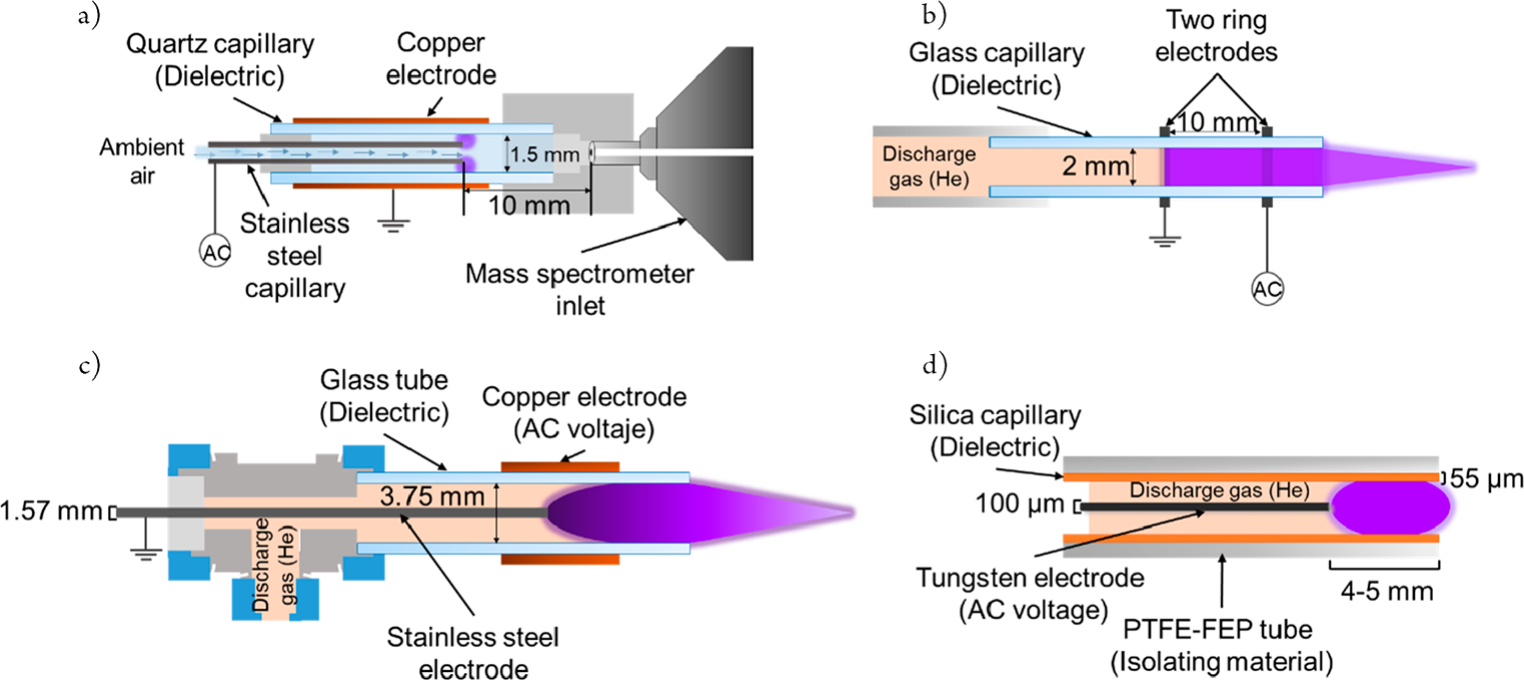
Diagrams of the dielectric barrier discharge ion sources: (a) active capillary plasma ionization (ACaPI), (b) dielectric barrier discharge ionization (DBDI), (c) low temperature plasma (LTP), and (d) flexible microtube plasma (FμTP).

In dielectric barrier discharges, a high voltage alternating waveform is applied between two electrodes that are separated by an insulator (usually glass), resulting in a plasma that is highly versatile in terms of operating pressure, geometric configuration, size, and density. DBD plasmas can be readily generated at ambient pressures and near room temperature using many different geometric configurations of the electrodes,^8^ dielectric material, gases,^9,10^ and high voltage pulsing sequences.^11,12^ For example, since DBD ionization (DBDI) was first reported in 2007 as an ion source for ion mobility spectrometry by Franzke and co-workers (Figure 1),^13^ several additional DBD-based ion sources have been developed, including low-temperature plasma (LTP),^14^ active capillary plasma ionization (ACaPI),^15^ and most recently flexible microtube plasma (FμTP) (Figure 1).^10^ DBD-based ion sources can be used to enhance the sensitivity and detection limits of liquid chromatography mass spectrometry for the detection of both polar and nonpolar compounds (e.g., amino acids and perfluoroalkanes)^16,17^ and can be readily integrated with solid-phase microextraction^18^ and complementary spray- and laser-based ionization methods.^19,20^ Moreover, DBD-based ion sources can be used to image the chemical profiles on surfaces^21^ or to passively sample volatile organic compounds from human breath with very high sensitivity.^22^

Ideally, an ambient ionization source should generate intact ions with minimal fragmentation. The fragmentation of ions in ambient ionization can limit sensitivity, increase spectral complexity, and hinder the assignment of ions and the interpretation of spectra. Thus, the extent of heating during ion formation and transfer is one the most fundamental characteristics of an ion source. Thermometer (TM) ion-based methods are well-established for characterizing the extent of energy deposited during ion formation^23−25^ and storage^26^ and as a result of ion activation.^27^

TM ions have been effectively used to assess the extent of ion heating for many different ion sources including electron ionization,^28^ electrospray ionization (ESI),^29,30^ secondary ion mass spectrometry (SIMS),^31^ laser-desorption,^32,33^ pulsed ion desorption,^31^ and some plasma-based ion sources including direct analysis in real-time (DART),^34,25^ ACaPI,^12^ and APCI.^35^ The “survival yield” (SY) method is widely used to estimate the extent and distribution of internal energy deposition during ion formation.^36^ In this approach, a series of TM ions that are structurally related and fragment by unimolecular dissociation through a single dissociation pathway with well-established bond dissociation energies are used.^37^ Internal energy distributions can be obtained from the measured survival yields of the ions and the minimum energy required to dissociate the ions (bond dissociation energy or BDE values).

A well-known suite of thermometer ions is based on the para-substituted benzylpyridiniums, which fragment primarily by cleavage of the C–N bond. The bond dissociation energies (BDEs) of benzylpyridiniums (∼160 to ∼260 kJ mol^–1^) depend strongly on the extent that the substituent donates or withdraws electron density from the C–N bond. More recently, benzylammonium thermometer ions were developed.^24^ In comparison to the benzylpyridiniums, benzylammonium thermometer ions have the advantages of fragmenting more readily owing to lower BDEs (∼100 to 200 kJ mol^–1^), ready availability from major commercial suppliers, and compatibility with a broader range of ion sources.

Here, we investigate the extent of ion heating by four major DBD-based ion sources (LTP, FμTP, ACaPI, and DBDI) using benzylammonium thermometer ions. The extent of internal energy distribution by the four DBD-based ion sources were “softer” than APCI. In addition, the extent of ion heating by ACaPI is substantially lower than that for the other DBD sources in their conventional orthogonal configurations and similar to ESI. However, the use of an alternate on-axis configuration can dramatically reduce the internal energy deposition by use of LTP and DBDI such that labile intact ions with low BDEs can be detected with minimal fragmentation. This is the first comprehensive survey of the extent of internal energy deposition during ion formation by a range of dielectric barrier discharge ionization sources.

## Materials and Methods

### Chemical and Solvents

LC-MS grade water and methanol were from Merck (Darmstadt, Germany). Five benzylammonium ions: 4-methoxy benzylamine (*p*OCH3), 4-methyl benzylamine (*p*CH3), 4-fluoro benzylamine (*p*F), benzylamine (*p*H), and 4-(trifluoromethyl) benzylamine (*p*CF3) were purchased from Fisher Scientific (Madrid, Spain). For all the internal energy distribution experiments, the sample solutions contained a mixture of 100 μM of each benzylamine in 50:50 methanol/water. Table 1 shows the *m*/*z* values of the precursor and fragment ions and the BDE of the C–N bonds for the thermometer ions. The BDE energies were previously reported by Stephens et al.^24^ and are based on *ab initio* electron structure calculations that were benchmarked to experimental data. Solutions of cholesterol, phenylalanine, and imidacloprid, purchased from Sigma-Aldrich (Madrid, Spain), were prepared at 10 μM concentration in 50:50 methanol/water for comparing the signal-to-noise ratios of the DBD-based ion sources.

**Table 1:** Mass-to-Charge (*m/z*) of the Parent and Fragment TM Ion of the Five Benzylammonium Ions and the Bond Dissociation Energies (BDEs) of the C–N Bond

TM ion	Precursor *m*/*z*	Fragment *m*/*z*	BDE (kJ mol^–1^)
pOCH_3_	138.1	121.1	105.8
pCH_3_	122.1	105.1	139.7
pF	126.1	109.1	152.8
pH	108.1	91.1	163.4
pCF_3_	176.1	159.1	159.1

### Mass Spectrometry

All the experiments were performed using a Thermo Finnigan LTQ linear ion trap mass spectrometer (Thermo Scientific, San José, CA, USA). Unless otherwise specified, the selected experimental conditions for the experiments comparing internal energy distributions (IED) and signal-to-noise (S/N) are shown in Table S1. The samples were injected using a syringe pump at 10 μL min^–1^ flow rate and vaporized using an Ion Max Vaporizer probe for APCI analysis (Thermo Scientific, San José, CA, USA) to evaluate comparable conditions that are used in liquid chromatography–MS measurements. The flowing solutions were vaporized using a heated capillary sheath set to 225 °C using a sheath flow (30 a.u.), auxiliary gas (5 a.u.), and sweep flow (5 a.u.) of N_2_(g). For ESI, the solutions were infused at the same rate into an Ion Max ESI probe (Thermo Scientific, San José, CA, USA) using an ESI spray voltage of 5 kV and sheath gas flow rate of 8 L min^–1^.

### Plasma-Based Ion Sources

Five plasma-based ion sources were evaluated. For ACaPI experiments, a homemade ion source, described elsewhere, was used (see Figure 1a).^12^ A modified version of the ACaPI (mACaPI) was used to perform experiments ensuring the sample introduction directly through the halo part of the plasma (rather than in the center of the halo-plasma) as shown in Figure S1 (refer to SI for full details). The DBDI (see Figure 1b) is a ring-to-ring DBD consisting of a glass capillary with a two annular electrodes separated by 10 mm, as previously described.^38^ For LTP, we used a home-built probe previously reported,^14^ that consists of a glass tube with an internal diameter of 3.75 mm surrounded by an outer copper high-voltage electrode with an inner, axial stainless steel grounded electrode (see Figure 1c). The FμTP used is a monoelectrode discharge operated by alternative current; the ion source configuration is shown in Figure 1d.^10^ For APCI, a needle (Thermo Scientific, San José, CA, USA) was used to generate a corona discharge (5 μA).

To apply high-voltage alternating currents for generating dielectric barrier discharges, two different power supplies were used. For DBDI, ACaPI, and FμTP, a homemade high-voltage, square wave power source was used with a maximum voltage of 3.5 kV and rates of voltage rise of ∼60 V ns^–1^. For LTP, the AC voltage was provided by a second custom-built power supply with a higher maximum voltage (5 kV) by coupling a sinusoidal waveform generator to a power amplifier and an automobile engine ignition coil.^14^ The operating conditions were selected based on previous optimization (Table 2).

**Table 2:** Operational Conditions for the Four DBD-like Ion Sources

Ion source	Voltage (kV)	Duty cycle	Helium flow (mL/min)	AC generator frequency (kHz)
ACaPI	1.8	50/50	-	20
DBDI	2.75	50/50	150	20
FμTP	1.5	50/50	75	20
LTP	7.2	50/50	400	2.7

**Figure 2:**
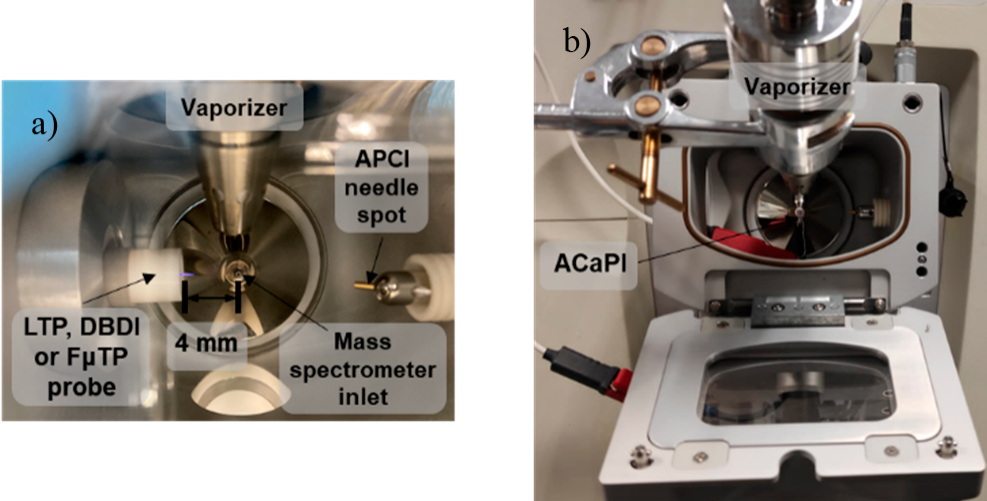
Photographs of the plasma sources in their standard configurations. (a) LTP, DBDI, and FμTP probes in an orthogonal orientation relative to the capillary entrance to the MS. (b) ACaPI in which the plasma flow is directed on axis to the capillary entrance to the MS.

The DBDI, FμTP, and LTP were used in two different configurations that were either orthogonal (orthog) or on-axis (on ax) with the capillary entrance to the MS (Figure 2). The latter configuration is unconventional for these specific ion sources. For the use of DBDI, FμTP, and LTP in their orthogonal configurations, the plasma sources were encased by Teflon holders, and the axes of the plasma streams were directed 90° to the axis of the inlet capillary (Figure 2). For the use of DBDI, FμTP, and LTP in their axial configurations, the plasmas were directed on axis to the inlet of the MS. In this case, the plasma probes were mounted on an *x*, *y*, *z* manual linear stage to control their position with respect to the mass spectrometer inlet. Some of the key dimensions for the different configurations are shown in Figure 2. For ACaPI, the vaporizer outlet was repositioned to accommodate the length of the ACaPI electrodes and inlet (Figure 2b). The distance and angle between the vaporizer to the inlet of the ACaPI source were adjusted to be essentially the same as the distance and angle between the vaporizer and the inlet to the MS for DBDI, FμTP, and LTP.

## Results and Discussion

### Internal Energy Deposition

**Figure 3:**
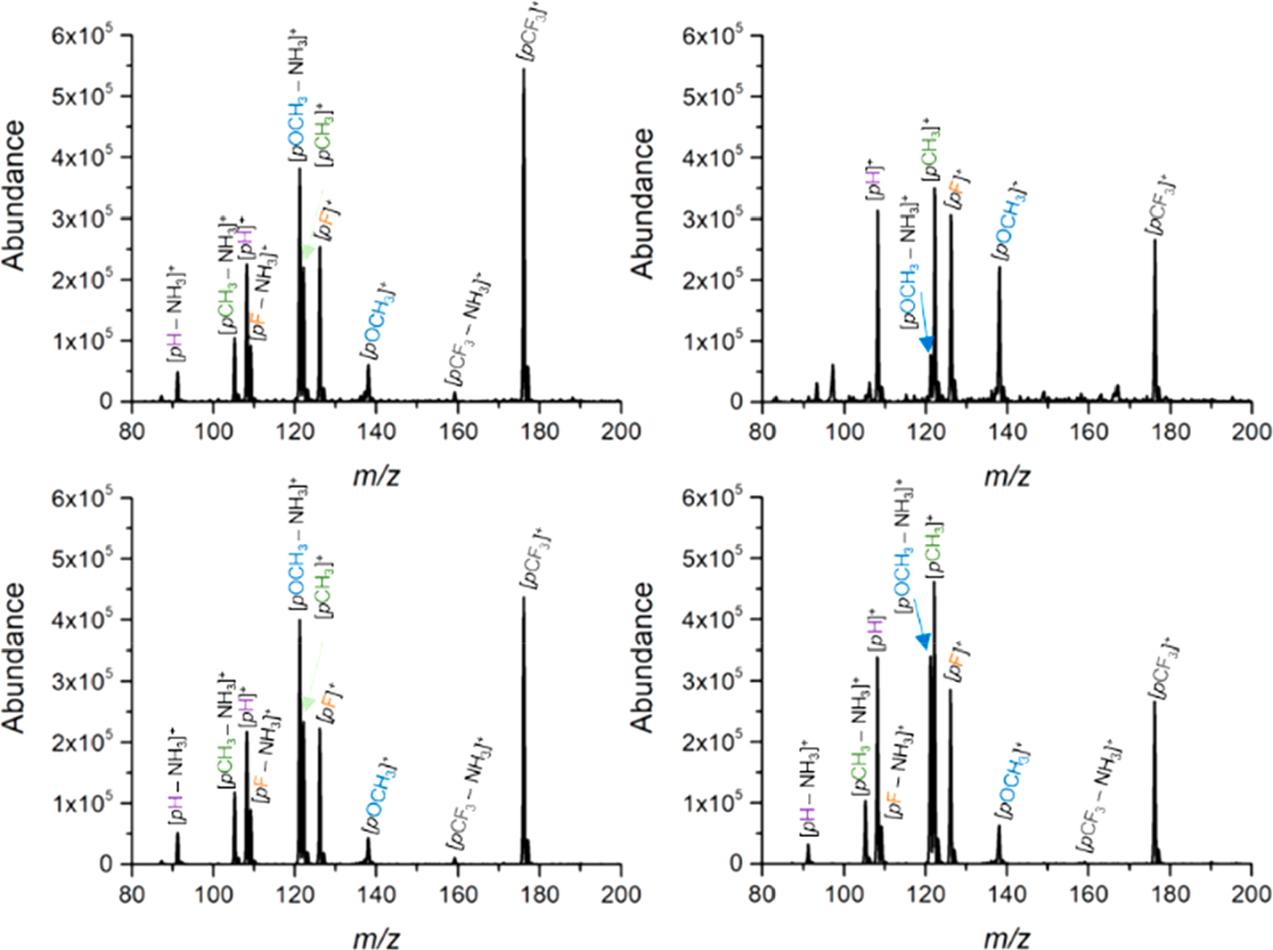
Representative DBD-based mass spectra of a mixture of five benzylamines obtained using (a) DBDI Orthog, (b) ACaPI, (c) FμTP Orthog, and (d) LTP Orthog. The precursor benzylammonium thermometer ions and their corresponding product ions are indicated.

**Scheme 1:**
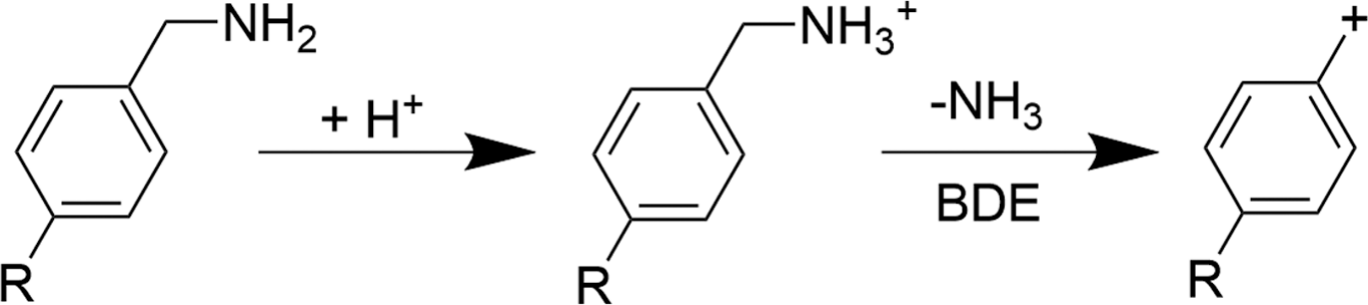
Formation and Fragmentation of Benzylammonium TM Ions

**Figure 4:**
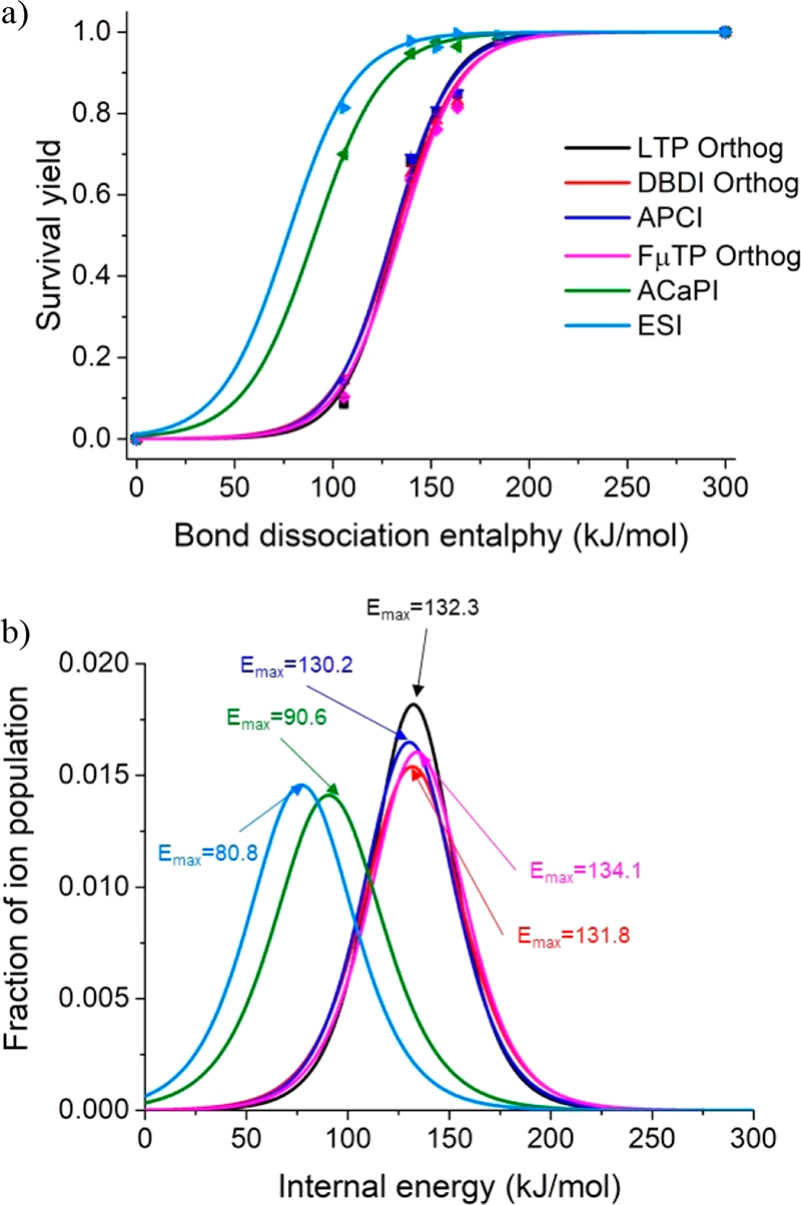
Comparison of the plasma-based ion sources using benzylammonium thermometer ions. (a) Survival yield vs BDE curves and (b) internal energy distributions. The *E*_max_ values (kJ mol^–1^) are indicated for each ion source.

Representative mass spectra of a mixture of five benzylamines obtained using the different DBD-based ion sources are shown in Figure 3. The five benzylammonium ions undergo unimolecular fragmentation via a single pathway that results in the loss of neutral NH_3_ (−17 Da) (Scheme 1). The relative extent of ammonia loss increases as the bond dissociation enthalpies of the C–N bond of each ion decreases. For example, the survival yields obtained for *p*OCH3, *p*CH3, *p*F, *p*H, and *p*CF3 are plotted as a function of the bond dissociation enthalpies for five plasma-based ion sources and ESI in Figure 4a. In general, the survival yields for each thermometer ion were comparable for APCI, LTP, DBDI, and FμTP. Moreover, the linear regression best-fit sigmoid curves to the SYs vs BDEs data for each of these four ions sources had inflection points and slopes that were within 2.9% and 17% of each other, respectively (Table S2). In stark contrast for ACaPI, the survival of the thermometer ions (with BDEs < 160 kJ mol^–1^) for ACaPI were substantially higher than for APCI, LTP, DBDI, and FμTP. For example, the survival yield of 4-methoxy-benzylammonium (BDE of 106 kJ mol^–1^) increased from ∼9–14% for APCI, LTP, DBDI, and FμTP to 70% for ACaPI. To obtain the internal energy distributions for each ion source under these conditions, the derivative of the best fit sigmoid curves to the SY vs BDE data were obtained (Figure 4b), consistent with previous studies.^39^ The median of the internal energy distribution for ACaPI was shifted to a value that was over 40 kJ mol^–1^ lower than the distributions for APCI, LTP, DBDI, and FμTP. Moreover, the latter four plasma-based ion sources had internal energy distributions with median values that were within ±3.9 kJ mol^–1^ of each other. These data indicate that ACaPI can be substantially “softer” than the other four ion sources and comparable to a soft ion source such as ESI (Figure 4b) under these conditions.

**Figure 5:**
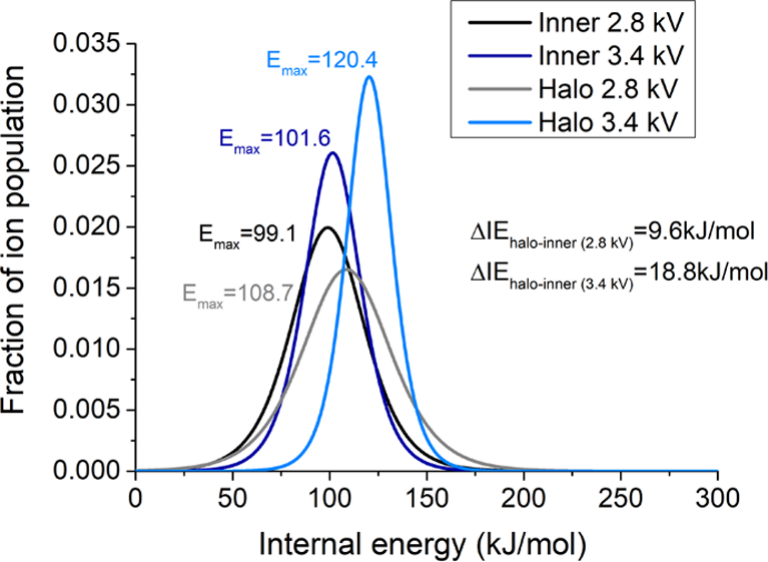
Internal energy distributions of mACaPI ion source when operated at 2.8 kV and 3.4 kV. The TM ions were introduced either directly through the halo of the plasma (Halo) or the inner electrode (Inner) in the center of the plasma ring. The *E*_max_ values (kJ mol^–1^) are indicated.

The type of plasma, the time the ions spend in the plasma, and the transfer of ions to the inlet of the MS can all potentially affect the internal energy distributions. The samples were vaporized at 225 °C in all cases, so the extent of internal energy resulting from the vaporization step should be comparable between all sources. In ACaPI, a halo-plasma is formed between the outside walls of the inner electrode and the quartz inner surface (Figure 1a).^9^ Thus, analytes should pass through the middle of the halo-plasma, and exposure to the “halo” of the plasma (where the plasma density is lower) should be relatively minimal (Figure 1a), analogous to a circus lion jumping through a ring of fire. To investigate the extent that ions are heated by passing directly through the plasma portion of the ring as opposed to through the center of the plasma halo, the ACaPI was modified to introduce benzylamines into the plasma halo rather than through the halo (Figure S1). The benzylamines were vaporized at 60 °C and introduced into the halo-plasma. The mACaPI ion source requires higher voltages (2.8 kV) to maintain stable plasma due to greater distances between electrodes and quartz. Internal energy distributions and *E*_max_ were slightly higher (10%) compared to the original ACaPI (Figure 5). Direct introduction of TM ions into the halo-plasma region resulted in a noticeable increase in *E*_max_ and internal energy deposited in ions. Increasing the voltage to 3.4 kV resulted in a harsher halo-plasma with an *E*_max_ of 120.4 kJ mol^–1^. In normal ACaPI, lower internal energy deposition can be attributed to reduced interaction between thermometer ions and the plasma by passing through the center of the plasma ring. That is, the other plasma ion sources form a plasma plume (APCI) or plasma-jets (FμTP, LTP, and DBDI), and the TM ions likely interact with a more reactive and energetic portion of the plasma regions, promoting higher internal energy deposition under these conditions (see below).

### Vaporization

Benzylammonium TM ions can be deprotonated in gas-phase collisions with neutral molecules, unlike nonvolatile benzylpyridinium thermometer ions. Such reactions can potentially affect the measured survival yields. Thus, the influence of the solvent composition on the internal energy distributions for benzylammonium ions was evaluated.^25^ Different ratios of methanol and water were used as the nebulizing solution because the gas phase basicity of each solvent is substantially different (725 and 660 kJ mol^–1^ for methanol and water, respectively). The solution composition had a negligible effect on the internal energy distributions for DBDI and a minor effect for ACaPI under these conditions (Figure S2). For example, increasing the concentration of methanol from 10% to 90% v/v shifted the *E*_max_ toward slightly higher energies (a difference of <8% corresponding to 6.7 kJ mol^–1^).

The solution vaporization temperature was evaluated as potential source of TM ion fragmentation.^23^ As shown in Figure S3, the temperature of vaporization had a relatively minor effect on the internal energy distributions obtained for the plasma-based ion sources. The *E*_max_ values increased by 5% for ACaPI as the vaporization temperature increased from 175° to 275°. The *E*_max_ values for the other ion sources also increased by between 7.5% and 9.5% as the temperature was increased from 175 to 275 °C. Previous results from thermometer ion experiments for direct analysis in real time (DART) MS indicated that the heated DART discharge gas had a minor effect on internal energy distributions.^25^ In this current work, the use of the vaporizer alone is essentially “thermospray”, which is basically electrospray ionization but without the electrification of the analyte solution.^40^ The heated nitrogen can facilitate desolvation of the droplets and promote ionization. However, the ion signal obtained by use of the vaporizer alone was exceedingly low. For example, the most labile TM ion (protonated *p*OCH3; *m*/*z* 121) was detected with an abundance that was at least 3 orders of magnitude lower than for the plasma ionization methods (Figure S3f). However, for the thermometer ions that were formed in sufficient abundance to detect both the precursor and fragment ions by use of thermospray, *p*OCH3 and *p*CH3, the survival yields were 2% and 64%, respectively. In contrast, the survival yields for these same ions that were obtained using ACaPI (70% and 90%) were substantially higher. Moreover, the vaporizer temperature also had minimal impact on the survival yield obtained for these ions.

### Effects of Voltage

The voltage supplied to ignite and sustain the plasma is a key parameter that can potentially affect the extent of ion heating. The internal energy distributions for ACaPI, DBDI, and FμTP were studied over a range of voltages that were sufficient to form ions that could be readily detected. In Figure S4, the resulting *E*_max_ values are plotted as a function of the applied voltage. For all three ion sources, the *E*_max_ values did not depend strongly on the applied voltages over a 1 kV working range. These data indicate that the applied voltage does not significantly affect the extent of internal energy deposition under these conditions.

### Effects of the Discharge Gas

The presence of highly energetic metastable discharge gas atoms and excited ions, atoms, or molecules play a role in plasma ionization mechanisms, which could potentially increase the likelihood of analyte fragmentation.^41^ In this study, two different discharge gases were used in DBD-based ion sources: helium for DBDI, FμTP, and LTP and air for ACaPI. Helium is commonly used as a discharge gas due to its low breakdown voltage and the high energy metastable atoms produced during the discharge, which promote the formation of reactant and radical ions. However, the short lifetime of the metastable atoms in the open air was found to be insufficient to cause significant fragmentation or direct Penning ionization of analytes in ambient mass spectrometry.^42,43^ A modified setup was built to evaluate the discharge gas effect in ACaPI. The results of this evaluation are shown in Figure S5. The benzylammonium ions were evaporated at 60 °C and different drag gases (helium, synthetic air, and ambient air) were used to transport and operate the ACaPI ion source. The internal energy distributions of ACaPI operated with helium, synthetic air, and ambient air were similar and within ±3.1 kJ mol^–1^ for the evaporative setup (Figure S5b). The minimal effect of the discharge gas on the ion source internal energy was also explored using FμTP. Three different discharge gases, helium, argon, and synthetic air, showed similar *E*_max_ values: 134.2, 135.6, and 137.6 kJ mol^–1^, respectively (Figure S5c). The extent of ion heating did not depend on the discharge gas for DBD-based ion sources in ambient mass spectrometry.

### Orthogonal vs Axial Configuration

**Figure 6:**
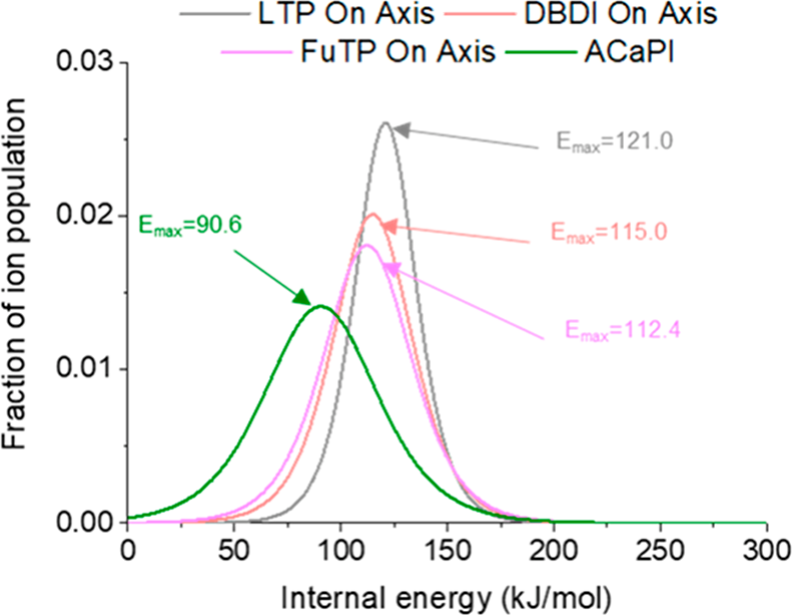
Internal energy distributions for LTP (gray trace), DBDI (light red trace), FμTP (pink trace), and ACaPI (green trace) when the plasma jets are oriented on axis with respect to the mass spectrometer inlet. The *E*_max_ values (kJ mol^–1^) are indicated.

Given that the vaporizer temperature, capillary inlet temperature, and plasma voltages had minimal to no effect on the *E*_max_, the effects of using different orientations of the vaporizer and plasma sources were investigated. To reduce the interactions of the TM ions with the higher density portion of the plasma jets for DBDI, FμTP, and LTP, the plasma jets were oriented on-axis with the capillary entrance to the MS, and the sample vaporizer was positioned to inject the analytes between the plasma jet and the capillary entrance to the MS (Figure S6); i.e., the analytes were injected such that they do not directly interact with the energy densest portion of the plasma jets (visible glowing and excited species emission). The extent of internal energy distributions for on axis DBDI, FμTP, and LTP are shown in Figure 6, in addition to that for ACaPI as a reference. The *E*_max_ values were shifted from around ∼130 kJ mol^–1^ for the orthogonal configuration to 112, 115, and 121 kJ mol^–1^ for the on axis configurations of FμTP, DBDI, and LTP, respectively. Thus, by changing the orientation, the extent of energy deposition by use of DBDI and FμTP could be reduced substantially (up to 16%), and that for LTP could be reduced by about 8.5%. Moreover, the *E*_max_ values for on-axis FμTP and DBDI were only ∼22–25 kJ mol^–1^ higher than that for ACaPI. In addition, by increasing the axial distance in between the plasma probe and the mass spectrometer inlet the average internal energies further decreased (Figure S7). For example, by increasing the distance of the outlet of the FμTP jet to the capillary entrance of the MS from 8 mm to 20 mm, the *E*_max_ decreased from 124 to 112 kJ mol^–1^. For on axis DBDI, moving the position from 15 to 25 mm from the capillary entrance reduced the *E*_max_ from 120 to 115 kJ mol^–1^. In contrast, this effect was more minor when the probe was positioned orthogonally (Figure S8). These data indicate that internal energy deposition can be reduced by use of the axial configuration. Overall, these data indicate that the extent of ion fragmentation could be reduced substantially by minimizing the extent of direct interactions with the plasma jets.

### Absolute Ion Abundance

**Figure 7:**
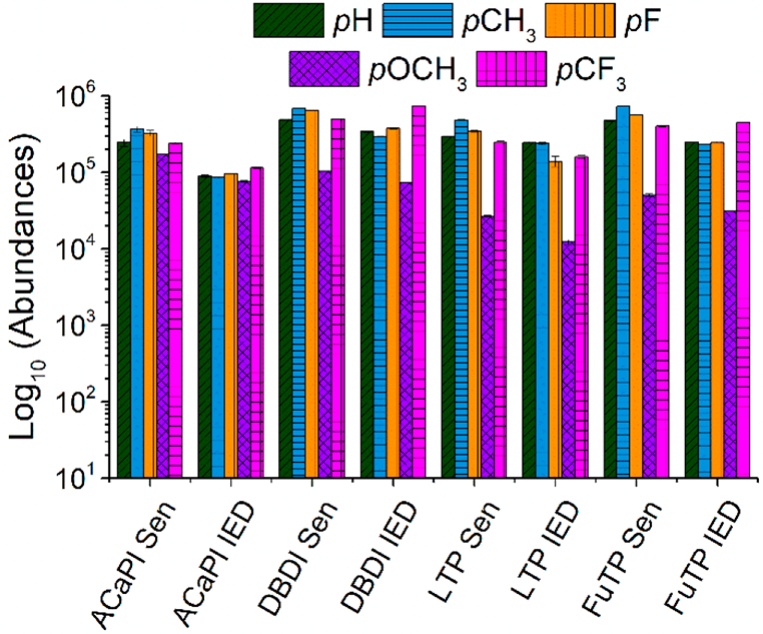
Ion abundances (log_10_ scale) for the five intact thermometer ions obtained using ACaPI, DBDI, LTP, and FμTP ion sources. Two different mass spectrometer experimental conditions were used: sensitivity (Sen) and internal energy distributions (IED) optimized conditions, as described in the Methods and Materials.

The ion abundances for the five intact benzylammonium ions (*m/*z values of 108.1, 122.1, 126.1, 138.1, and 176.1) were compared for the DBD-based ion sources. These data were obtained in triplicate on the same day. Generally, the abundances were comparable between DDBI, LTP, and FμTP for these ions, while ACaPI resulted in lower abundances (Figure 7). A similar general trend across the ion sources for the abundances of other compounds (phenylalanine, cholesterol, and imidacloprid) was also obtained (Table S3). Optimizing the ion transmission parameters of the mass spectrometer to increase the abundances of these ions resulted in similar abundances for these ions as were used for the experiments used to measure internal energy distributions. Overall, these data indicate that under these conditions, ACaPI can be considered the “softest” of these plasma ion sources with moderately high sensitivity.

## Conclusions

The use of DBD in an active capillary configuration (ACaPI) can substantially lower the extent of internal energy deposition in DBD sources to ensure that intact ions with very labile bonds can be readily detected with comparably high sensitivity. For example, the average internal energy distribution for ACaPI was over 30% lower than other DBD-based sources (DBDI, LTP, and FμTP) and a corona discharge-based source (APCI). The extent of ion fragmentation depends strongly on the extent of interaction between the dense regions of plasma (halo or jet) and the analytes. For example, by decreasing the distance between the DBD plasma jets further from the source of analyte introduction, the extent of ion internal heating was decreased for DBDI, LTP, and FμTP. In ACaPI, gaseous analytes are driven toward the capillary inlet to the mass spectrometer through the center of a DBD halo-plasma, which minimizes interactions between the plasma and the vaporized molecules to reduce the extent of energy deposited during ionization. The use of different discharge affects the extent of ion heating minimally. By configuring DBDI, LTP, and FμTP plasma jets on axis with the capillary inlet to the MS to minimize interactions between the vaporized analytes and the highly energetic regions of the plasma, the average energy deposited was reduced by 8.5%, 13.0%, and 16.0% for LTP, DBDI, and FμTP. This research is anticipated to facilitate the development of ultrabright plasma ion sources that are also soft and compatible with a broad range of analyte classes.

## Supporting Information Available

Detailed information and experimental results including the ACAPI design for halo plasma experiments, the influence of the solvent composition in the extent of internal energy deposited, the effect of the vaporizer temperature in the plasma-based ion sources internal energy distributions, the effect of the voltage on *E*_max_ for ACaPI, DBDI, and FμTP; the effect of influence discharge gases in DBD ion sources internal energy distributions; the schematic of the relative positions of LTP, DBDI, and FμTP ion sources, vaporizer and mass spectrometer inlet; the effect of the plasma-jet distance with respect to the mass spectrometer inlet in the axial configuration for FμTP, DBDI, and LTP, and in the orthogonal position for DBDI. Also, tables with the selected experimental conditions, the sigmoid curve fitting parameters are included and abundances and signal-to-noise ratios for the DBD-based ion sources evaluated (PDF)
