# Supplementary material for: Ion Heating in Advanced Dielectric Barrier Discharge Ion Sources for Ambient Mass Spectrometry
Source: J Am Soc Mass Spectrom. 2023 May 26;34(6):1145–52. doi: 10.1021/jasms.3c00087 (PMC10251516; doi:10.1021/jasms.3c00087)
Supplement: Supplementary file 1 — js3c00087_si_001.pdf [file js3c00087_si_001.pdf]

Supplementary material

Ion heating in advanced dielectric barrier discharge ion sources for ambient mass spectrometry

Marcos Bouza[1*], Ezaz Ahmed[2], Priscilla Rocio-Bautista[1], Sebastian Brandt[3], Joachim Franzke[3], Antonio Molina-Díaz[1], Juan F. García-Reyes[1] and William A. Donald[2*]

[1]Analytical Chemistry Research Group, Department of Physical and Analytical Chemistry, University of Jaén, Campus Las Lagunillas, 23071 Jaén, Spain.

[2]School of Chemistry, University of New South Wales, Sydney, NSW 2052, Australia.

[3]ISAS—Leibniz Institut für Analytische Wissenschaften, Bunsen-Kirchhoff-Str. 11, 44139 Dortmund, Germany.

[*]Corresponding authors: Marcos Bouza, email: mbouza@ujaen.es, phone: +34 953 21 2758,
William A. Donald, email: w.donald@unsw.edu.au, phone: +61-2-9065-9401.

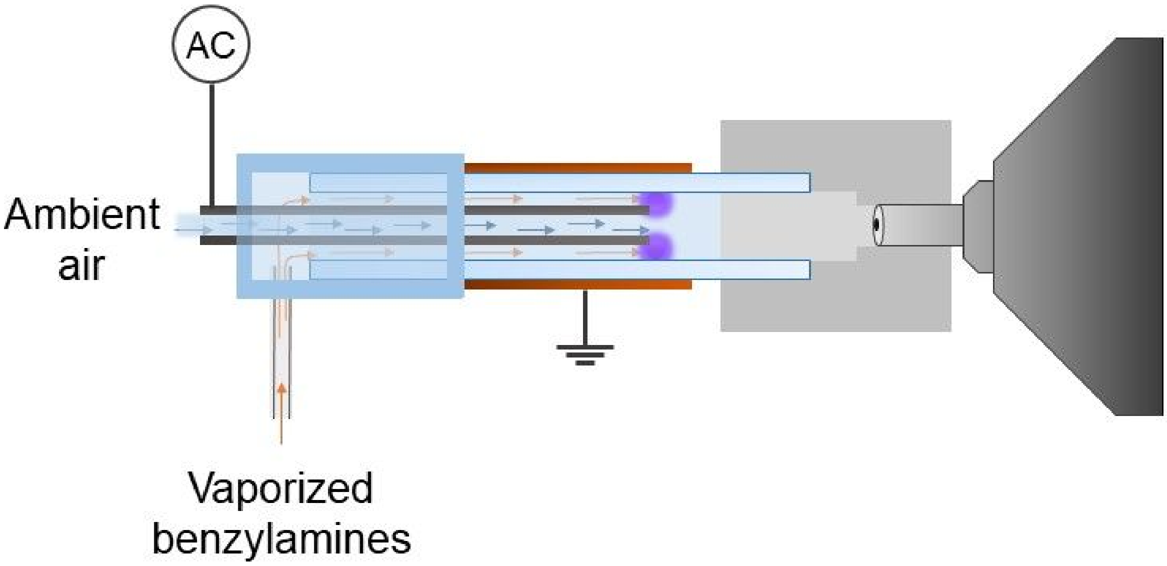

Figure S1. Diagram of ion source to introduce the benzylamines through the DBD plasma of the ACaP ion source rather than in the center of the halo plasma. The inner electrode has an outer diameter of 0.907 mm and inner diameter of 0.603 mm.

This configuration permits the analytes to go through the plasma and directly interact with the more energetic portion of the plasma. The vacuum from the capillary entrance to the MS ensures the supply of ambient air towards the inner electrode to sustain the plasma.

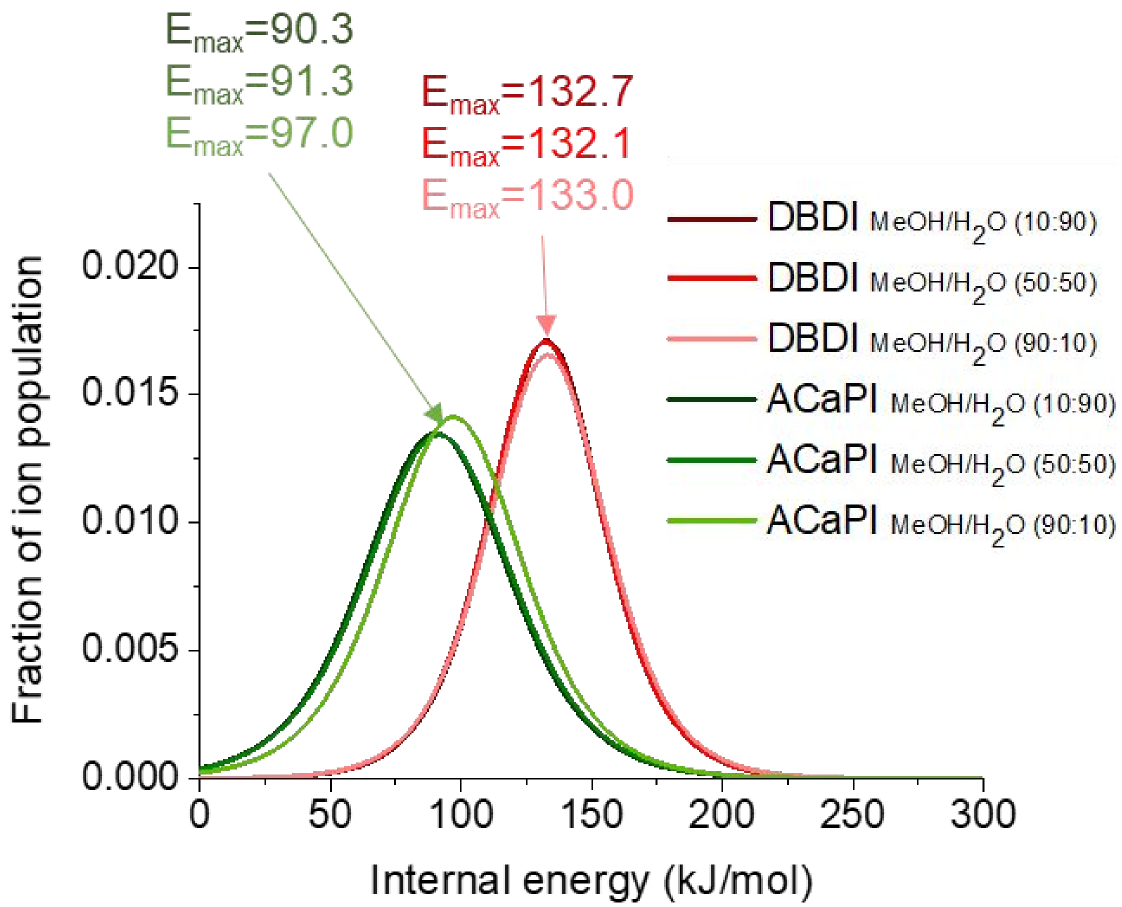

Figure S2. Effects of the solvent composition in the internal energy distribution for two representative plasma ion sources. Methanol and water mixtures of 10:90, 50:50 and 90:10 were evaluated for DBDI Orthog (red traces) and ACaPI (green traces).

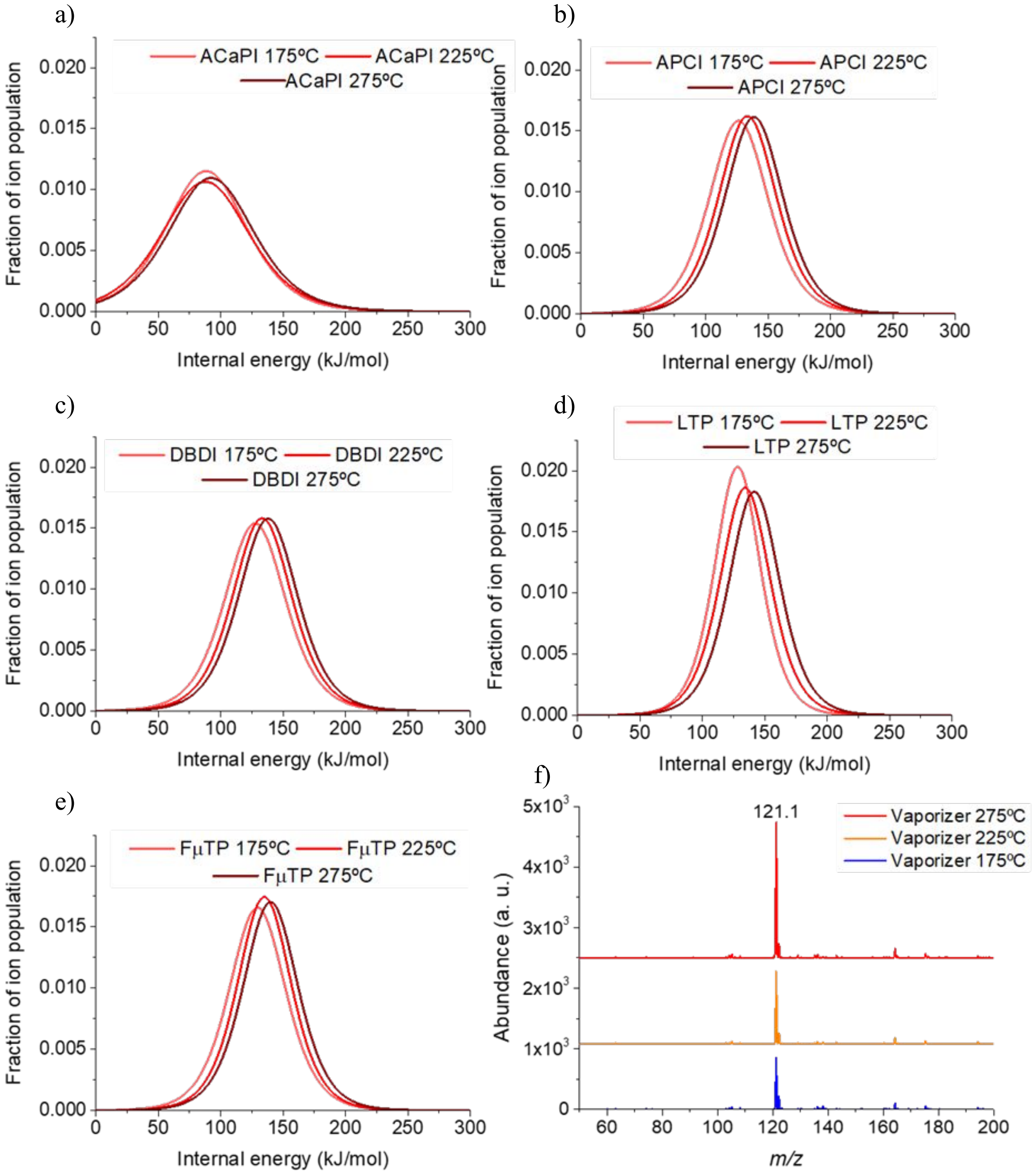

Figure S3. Effects of the vaporizer temperature (175º, 225 and 275ºC) in the internal energy distributions observed for the evaluated plasma-based ion sources: a) ACaPI, b) APCI, c) DBDI Orthog, d) LTP Orthog and e) FμTP Orthog. f) Thermospray vaporized TM ions.

The ion source and the in-source collision induced dissociation in the mass spectrometer first vacuum stages can favor different degrees of dissociation. The fragment ion of the most labile TM ion (*m/z* = 121.1) was observed. However, as observed in Figure S2f, the abundances was, at least, three order of magnitude lower compared to the plasma-based ion sources, having negligible contributions in the internal energy distribution.

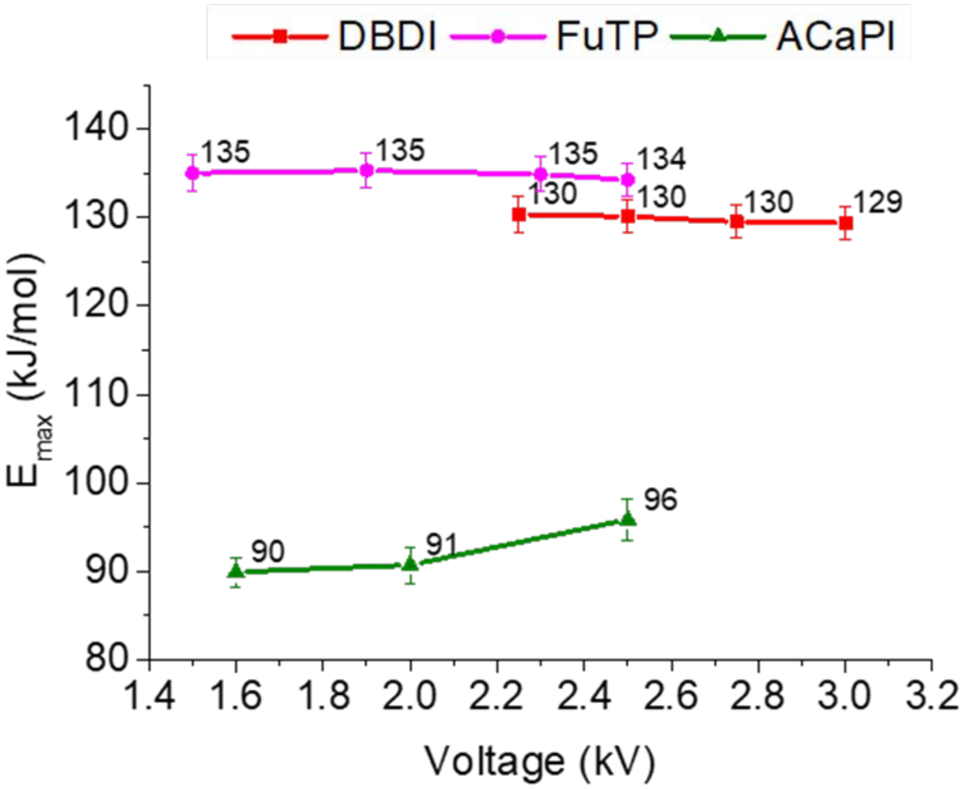

Figure S4. Average of the internal energy deposition (E_{max}) for a range of working voltages of ACaPI (black), DBDI (red) and FμTP (blue).

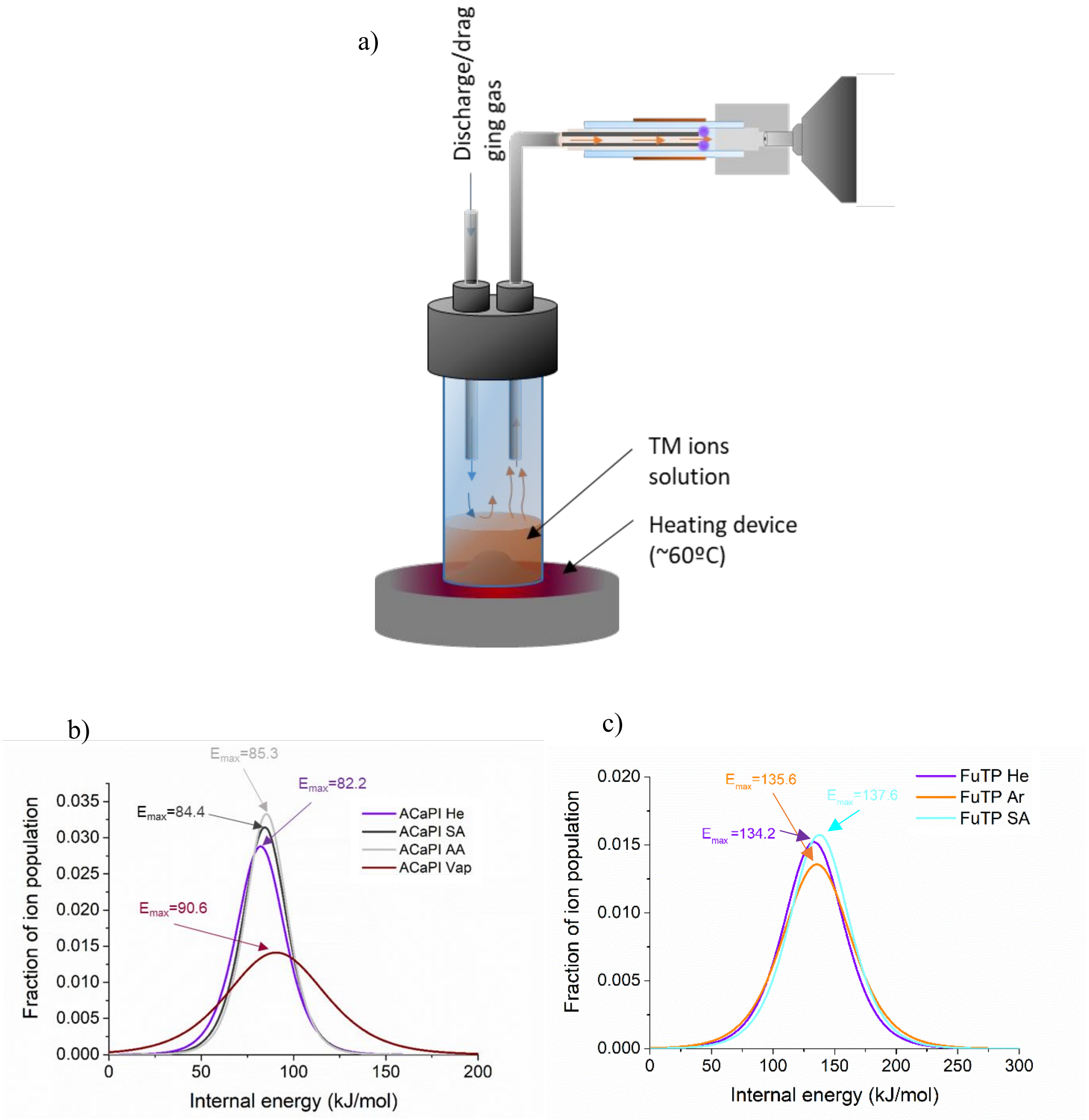

Figure S5. Internal energy distributions for different discharge gases. a) Scheme of the system used to introduce the evaporated TM ions in the ACaPI ion source. b) Internal energy distributions of different discharge gases (He, synthetic air (SA) and ambient air (AA)) used to help the transport of headspace-analyzed TM ions compared to the vaporized TM ions internal energy distribution (dark wine trace). c) FµTP for He, Ar and SA as discharge gases.

The built setup for the ACaPI analysis consists of a 20 mL vial with a two-inlet cap placed on a heater maintained at 60ºC. Five milliliters of the 100 µM solution was introduced in the vial. When a dragging gas was used (He and SA) a constant flow of the gas was supplied to drag the evaporated TM ions and maintain the discharge. On the other hand, the discharge gas inlet was open when the ACaPI was operated with ambient air. The atmospheric air, as well as the TM ions, were introduced in the discharge region helped by the mass spectrometer.

The miniaturized dimensions of FµTP enabled the operation with different discharge gases within the maximum operation range of the square-wave voltage supply used, 3.5 kV. The operation voltages were 1.5 kV for He and 3.3 kV for Ar and synthetic air. ACaPI was operate at 1.8 kV in all of the cases.

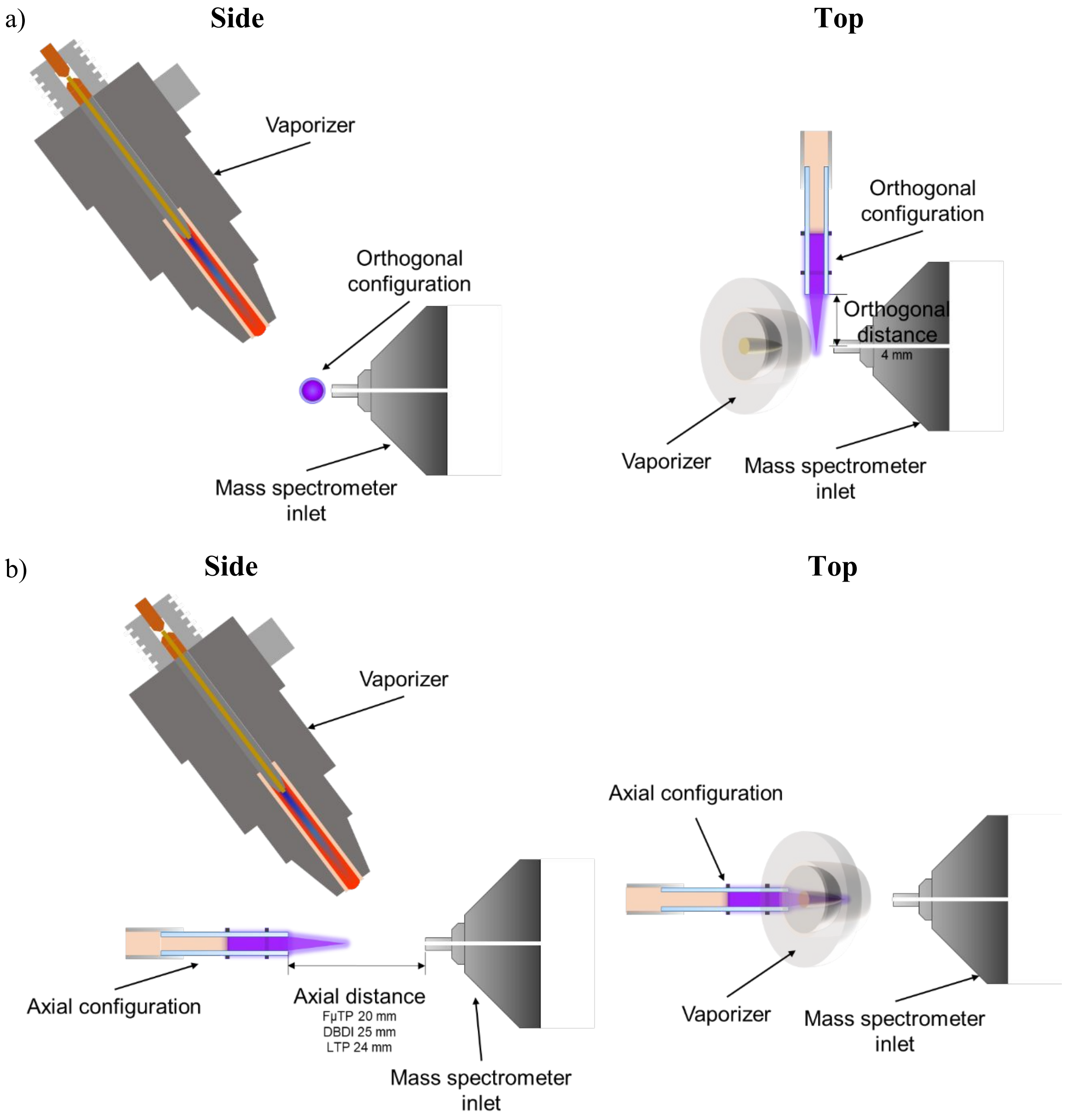

Figure S6. Schematic of the side and top view of the ion source-vaporizer-mass spectrometer configurations used in the present work for DBDI, FμTP and LTP; a) orthogonal, and b) axial (on-axis) configuration.

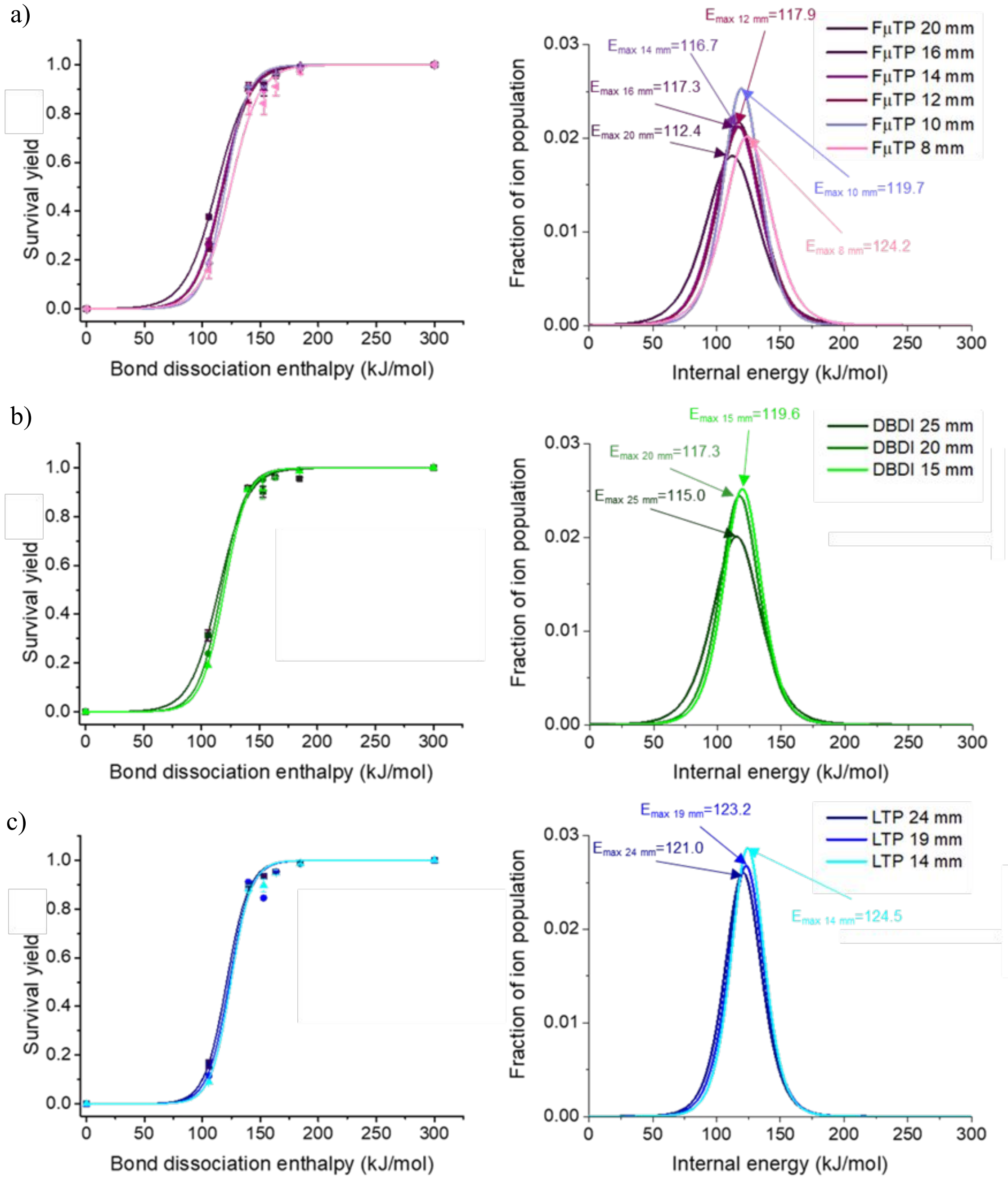

Figure S7. Effects of the axial distance (gap in between the ion source capillary and the mass spectrometry inlet). The breakdown dissociation curves (right) and the internal energy distributions (left) were obtained for different distances, according with the plasma nature and the plasma-jet length (LTP>DBDI>FμTP), for: a) FμTP, b) DBDI and c) LTP.

The on-axis distances were controlled using an *x, y,* z stage. Both, DBDI and LTP, did not show survival yield changes when the distances were shorter than 15 mm.

The small plasma volume of FμTP required from shorter distances (<12 mm) to promote noteworthy abundances (>1000 a. u.). However, the increased intensities also promoted a higher degree of fragmentation. In addition, when the probe was positioned at 8 mm with respect to the mass spectrometer inlet, we observed the decrease of the TM ions signals; the probe was partially blocking the vaporized molecules path towards the plasma-jet.

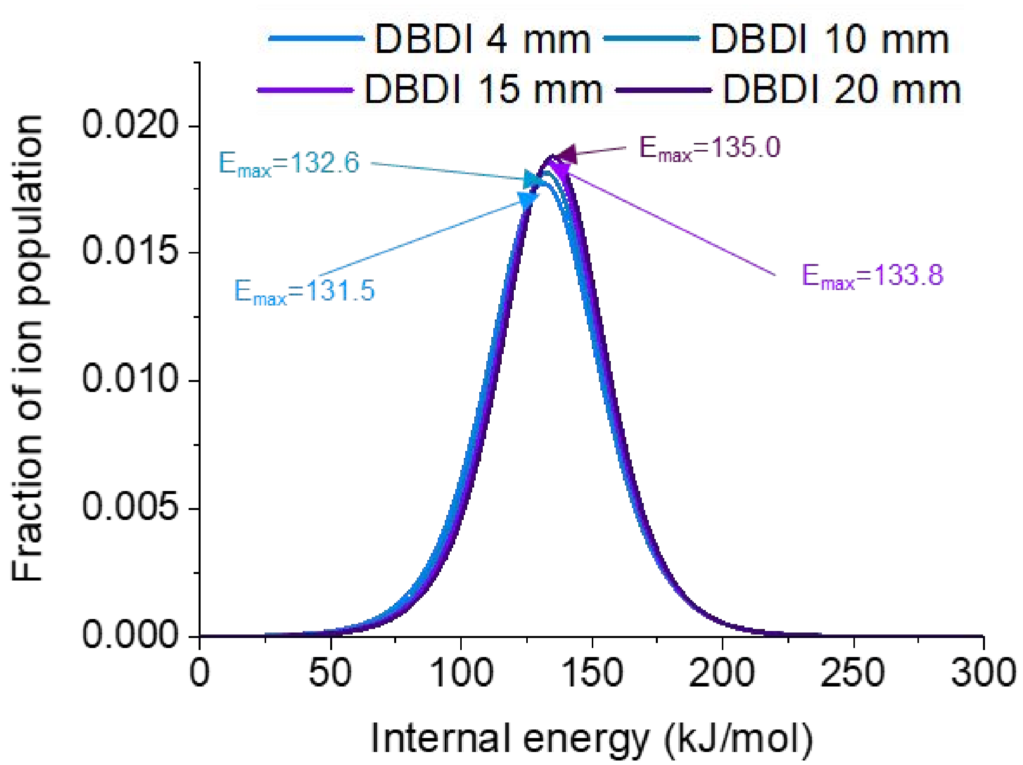

Figure S8. Effects of the distance of DBDI orthogonal plasma-jet with respect to the mass spectrometer inlet.

Table S1. Selected experimental conditions for the experiments comparing: a) internal energy distributions (IED) and b) signal to noise (S/N).

a)

**Parameter**	**Values**
Capillary temperature	150ºC
Capillary voltage	10 V
Tube lens voltage	60 V
Maximum ion trap injection time	200 ms
Number of microscans	2

b)

**Parameter**	**Values**
Capillary temperature	200ºC
Capillary voltage	10 V
Tube lens voltage	30 V
Maximum ion trap injection time	200 ms
Number of microscans	2

Table S2. Parameters obtained for the sigmoidal fitting of the five evaluated TM ions survival yields in Figure 3. Slope value in the table corresponds to the slope of the sigmoid curve at survival yield 0.5, calculated as $\frac{1}{4dx}$.

Ion source	x_0	Se	dx	Se	Slope
LTP	132.3	1.9	13.8	1.5	0.018
DBDI	131.8	1.5	16.2	1.2	0.015
APCI	130.2	1.6	15.2	1.3	0.016
FµTP	134.1	1.8	15.6	1.6	0.016
ACaPI	90.6	1.7	17.7	1.4	0.014

The sigmoidal fit was done using the following equation:

$$y = 1/(1 + \exp((x-x_0)/dx)) \qquad \text{equation 1}$$

being x_0 is the fitted modal average internal energy of the sigmoidal fit and dx the time constant.

Table S3. Abundances and signal-to-noise (S/N) ratios for the DBD-based ion sources. For all the evaluated compounds a 10 µM of each in 50:50 methanol/water were measured. The S/N was calculated integrating the same m/z range for 3 replicates of the analyte analysis and 3 replicates of the solvent blank in the same range (phenylalanine m/z=166.0-166.4, cholesterol m/z=369.0-369.8, and imidacloprid m/z=256.0-256.5).

	**Phenylalanine**	**S/N**	**Cholesterol**	**S/N**	**Imidacloprid**	**S/N**
**DBDI**	1.30E+04±5.22E+02	21.1	4.44E+05±5.56E+03	90.1	3.69E+05±9.06E+03	79.1
**FuTP**	1.47E+04±1.04E+02	23.8	5.44E+05±8.33E+04	135.8	4.77E+05±3.43E+04	96.9
**LTP**	1.73E+04±5.54E+02	26.5	6.46E+05±6.48E+04	221.3	4.63E+05±3.53E+04	85.4
**ACaPI**	5.30E+03±2.25E+03	7.4	1.68E+05±1.15E+04	91.9	1.58E+05±2.04E+04	69.6
